# Glycocalyx Impairment in Vascular Disease: Focus on Inflammation

**DOI:** 10.3389/fcell.2021.730621

**Published:** 2021-09-13

**Authors:** Jing Qu, Yue Cheng, Wenchao Wu, Lixing Yuan, Xiaojing Liu

**Affiliations:** ^1^Laboratory of Cardiovascular Diseases, Regenerative Medicine Research Center, West China Hospital, Sichuan University, Chengdu, China; ^2^Department of Cardiology, West China Hospital, Sichuan University, Chengdu, China; ^3^Public Laboratory of West China Second University Hospital, Sichuan University, Chengdu, China

**Keywords:** glycocalyx, endothelial cell, endothelial dysfunction, inflammation, inflammasome

## Abstract

The glycocalyx is a complex polysaccharide-protein layer lining the lumen of vascular endothelial cells. Changes in the structure and function of the glycocalyx promote an inflammatory response in blood vessels and play an important role in the pathogenesis of many vascular diseases (e.g., diabetes, atherosclerosis, and sepsis). Vascular endothelial dysfunction is a hallmark of inflammation-related diseases. Endothelial dysfunction can lead to tissue swelling, chronic inflammation, and thrombosis. Therefore, elimination of endothelial inflammation could be a potential target for the treatment of vascular diseases. This review summarizes the key role of the glycocalyx in the inflammatory process and the possible mechanism by which it alleviates this process by interrupting the cycle of endothelial dysfunction and inflammation. Especially, we highlight the roles of different components of the glycocalyx in modulating the inflammatory process, including components that regulate leukocyte rolling, L-selectin binding, inflammasome activation and the signaling interactions between the glycocalyx components and the vascular cells. We discuss how the glycocalyx interferes with the development of inflammation and the importance of preventing glycocalyx impairment. Finally, drawing on current understanding of the role of the glycocalyx in inflammation, we consider a potential strategy for the treatment of vascular diseases.

## Introduction

The glycocalyx is a general term for polysaccharide protein complexes covering the surface of vascular endothelial cells. As the skeletal structure of the endothelial cell surface, the glycocalyx is a key factor in the regulation of the fluid balance inside and outside of blood vessels and is closely related to vascular permeability (Jedlicka et al., 2020). Glycocalyx impairment is associated with many diseases, such as atherosclerosis, diabetes, and sepsis, all of which are related to chronic inflammation. Table 1 lists the main diseases known to be related to glycocalyx impairment. In recent years, there have been major advances in anti-inflammatory drugs used to treat diabetes, atherosclerosis, and sepsis. Among these drugs, interleukin-1 (IL-1) receptor antagonists have attracted much attention (Jamilloux et al., 2018). Statins are now commonly used for the treatment of atherosclerosis due to their cholesterol-lowering and anti-inflammatory effects (Horodinschi et al., 2019). Sepsis refers to a systemic inflammatory response syndrome caused by infection. The treatment of sepsis involves early circulatory resuscitation, as well as anti-inflammatory therapy (Huang et al., 2019). Although current anti-inflammatory treatments can alleviate the inflammatory response to some extent, they cannot restore endothelial dysfunction after glycocalyx impairment.

**TABLE 1:** Main diseases to glycocalyx impairment.

Diseases	References	Diseases	References
Systemic or local inflammation	Margraf et al., 2018	Sepsis	Iba and Levy, 2019; Fernández-Sarmiento et al., 2020
Diabetes mellitus	Dogné et al., 2018	Ischemia-reperfusion injury	van Golen et al., 2012; Abassi et al., 2020
Chronic and acute renal disease	Rabelink and de Zeeuw, 2015; Tarbell and Cancel, 2016	Atherosclerosis	Tarbell and Cancel, 2016; Mitra et al., 2017
Stroke	Zeng, 2017	Hypertension and pulmonary oedema	Collins et al., 2013; Mendes et al., 2020
Cancer	Kang et al., 2018; Buffone and Weaver, 2020	COVID-19	Yamaoka-Tojo, 2020a, b

Endothelial cells consist of a single layer of cells covering the vascular cavity. The vascular endothelium serves as the first barrier, thereby providing protection against the effects of inflammation. Damage to the glycocalyx layer is thought to be initial stage in the development of inflammation (Lupu et al., 2020). The glycocalyx is connected to the endothelium by backbone molecules, including proteoglycans and glycoproteins. These interact to form a network structure, with various plasma-derived and endothelial cell-derived soluble biological macromolecules incorporated into this network to form the basic structure of the glycocalyx (Jedlicka et al., 2020). Due to the location of the glycocalyx, the entire structure provides a barrier to water and solute transmission and acts as a bridge for interactions between blood circulating cells and endothelial cells. The glycocalyx also functions as a sensor of mechanical forces, and it protects against overactivation of cell surface receptors (Pillinger and Kam, 2017). However, the structure of the glycocalyx is extremely vulnerable, and inflammation, ischemia/reperfusion, hypervolemia, and vascular surgery can cause endothelial glycocalyx impairment. Such impairment causes a decrease in anticoagulants, an increase in endothelial permeability, enhanced migration of proinflammatory cells, impaired mechanical conduction, and endothelial nitric oxide (NO) synthase activity (Sieve et al., 2018). Oxidative stress plays an important role in the progression of endothelial dysfunction. It serves as an intermediate trigger, activating the NOD-like receptor pyrin domain-containing 3(NLRP3) inflammasome and aggravating the subsequent inflammatory cascade and endothelial dysfunction (Incalza et al., 2018). Damage to the glycocalyx layer leads to endothelial cell dysfunction. Vascular endothelial dysfunction aggravates the inflammatory response, which leads to a cycle of inflammation and endothelial dysfunction, with the inflammatory response further aggravating glycocalyx impairment.

In this review, we summarize recent advances in understanding of the effects of glycocalyx impairment, focusing on inflammation development. We discuss components of the glycocalyx in modulating the inflammatory process. We conclude by discussing preventing glycocalyx impairment might provide a strategy to interrupt the cycle of endothelial dysfunction and inflammation.

## Structure and Function of the Glycocalyx

**FIGURE 1:**
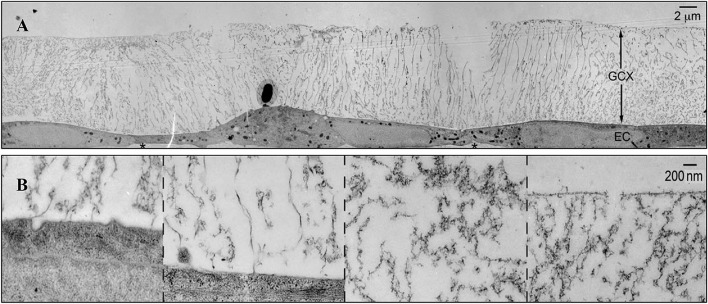
The structure of glycocalyx (Tarbell and Cancel, 2016). **(A)** Transmission Electron Microscope of glycocalyx preserved by ruthenium red and osmium tetroxide. **(B)** High-magnification image of glycocalyx.

**FIGURE 2:**
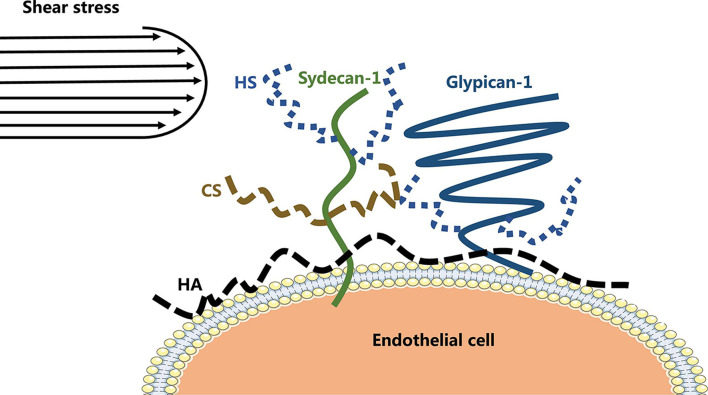
The structure of endothelial glycocalyx.

The vascular endothelial glycocalyx comprises a layer of villous polyglycoproteins with a composite structure that are located on the apical membrane of endothelial cells between the tube wall and blood (Figure 1). The endothelial glycocalyx serves as a natural dynamic barrier on the surface of these cells (Jedlicka et al., 2020). The main components of the endothelial glycocalyx are glycoproteins with sialic acid residues at the ends and proteoglycans with glycosaminoglycan (GAG) side chains. GAGs are linear heteropolysaccharides, which contain one molecule of hexosamine and one molecule of hexuronic acid. They are huge family composed of specific combinations of hexosamine and hexuronic acids (Curry, 2018). GAGs found on the surface of endothelial cells include heparan sulfate (HS), chondroitin sulfate (CS), and hyaluronic acid (HA). Two families of cell surface molecules (syndecans and glypicans) make up the core protein skeleton of endothelial glycocalyx (Aldecoa et al., 2020). Syndecan-1 combines with HS and CS, playing an important role in signal transduction. Glypican-1 binds to HS, which is directly anchored to a lipid raft structure rich in cholesterol and sphingolipids via C-terminal phosphatidylinositol (Figure 2). This structure plays a role in vesicle transport and signal transduction. The glycocalyx covers the surface of all vascular endothelial cells and serves an important function in the pathophysiology of blood vessels (Cosgun et al., 2020). The glycocalyx has three main functions: (1) It acts as a bridge for interactions between blood circulating cells and endothelial cells, (2) it acts as a selective permeable barrier for the blood vessel wall, and (3) it acts as a mechanical sensor of blood shear force (Cosgun et al., 2020).

## The Role of the Endothelial Glycocalyx Layer in Inflammation and Endothelial Dysfunction

The vascular endothelial glycocalyx layer is a central player in the inflammatory response. Lipowsky (2018) observed rapid shedding of vascular endothelial glycocalyx layer in a murine inflammation model and the release of inflammation mediators, such as reactive oxygen species (ROS), reactive nitrogen species, and tumor necrosis factor-α (TNF-α), which impaired the structural integrity of the glycocalyx, thereby affecting its function (Lipowsky, 2018; Uchimido et al., 2019; Gallagher et al., 2020). After the structure of the vascular endothelial glycocalyx is damaged, vascular endothelial cell intercellular adhesion molecule 1 (ICAM-1) and vascular cell adhesion molecule 1 (VCAM-1) are exposed. As a result, leukocytes in the blood circulation can adhere more easily to vascular endothelial cells. This process promotes the development of inflammation and endothelial dysfunction (Mulivor and Lipowsky, 2004; Vestweber, 2015; Bui et al., 2020; Liew et al., 2021). Therefore, glycocalyx shedding is an important factor in vascular endothelial dysfunction.

Endothelial dysfunction results in a reduction in the level of NO in blood vessels, which, in turn, leads to abnormal vascular function. Evidence suggests that the characteristics of endothelial dysfunction include weakened endothelial-mediated vasodilation, disturbed hemodynamics, impaired fibrinolytic ability, and excessive generation of ROS and oxidative stress (Gimbrone and García-Cardeña, 2016; Incalza et al., 2018; Cyr et al., 2020; Zuchi et al., 2020).

The inflammatory response is an important mechanism underlying the development and progression of endothelial dysfunction, and it plays a pivotal role in the pathological process of vascular diseases. Soeki and Sata (2016) showed that high-sensitivity C-reactive protein, an inflammatory marker, is associated with metabolic risk factors for cardiovascular diseases (Li et al., 2018). C-reactive protein can damage the vascular endothelium, resulting in a decrease in NO production by the vascular endothelium (Sproston and Ashworth, 2018). When an inflammatory reaction occurs in blood vessels, B lymphocytes, T lymphocytes, and mononuclear are activated. This leads to an increase in the production of IL-6 and TNF-α. The activities of IL-6 and TNF-α are interlinked, with TNF-α inducing the production of IL-6 and IL-6 stimulating the liver to increase the production of C-reactive protein or vice versa. Macrophages in atherosclerotic plaques, neutrophils, and monocytes in the blood synthesize TNF-α, which induces the release of TNF-α in the presence of arterial injury. TNF-α rapidly upregulates endothelial cell adhesion factors, which activate endothelial cells and inflammatory cell aggregation and lead to the release of inflammatory mediators (Sahibzada et al., 2017; Ng et al., 2018; Wang and He, 2020). TNF-α also regulates endothelial cell damage and remodels through the nuclear factor-κB (NF-κB) signaling pathway (Hayden and Ghosh, 2014; Blaser et al., 2016).

Reactive oxygen species and RNS released in the inflammatory response degrade HA, HS, and CS. ROS and RNS cause degradation of vascular endothelial glycocalyx by activating matrix metalloproteins (MMPs) and inactivating endogenous protease inhibitors (Rubio-Gayosso et al., 2006; van Golen et al., 2014). Proteases result in structural damage to the vascular endothelial glycocalyx. This damage, with the associated loss of the activity of various enzymes, including superoxide dismutase, antithrombin III, and thrombomodulin, as well as that of signaling molecules, results in weakening or loss of the barrier function of the endothelial glycocalyx layer (Kolářová et al., 2014; Sieve et al., 2018; Moore et al., 2021). This eventually leads to an imbalance in the enzymatic system, with endothelial barrier coagulation and antioxidant dysfunction. Most importantly, damage to the structure of the vascular endothelial glycocalyx via an inflammation reaction disturbs the mechanical stress transduction function of the glycocalyx. The latter leads to a series of pathological changes, including increased vascular permeability, edema, changes in the interactions between endothelial cells and white blood cells, and an imbalance in the coagulation and antioxidant systems, and decreased vascular tone (Yao et al., 2007; Chappell and Jacob, 2014). These changes further exacerbate endothelial dysfunction. Therefore, endothelial glycocalyx impairment is a crucial factor in the cycle of inflammation and endothelial dysfunction.

## Mechanism by Which the Endothelial Glycocalyx Layer Regulates Inflammation

**FIGURE 3:**
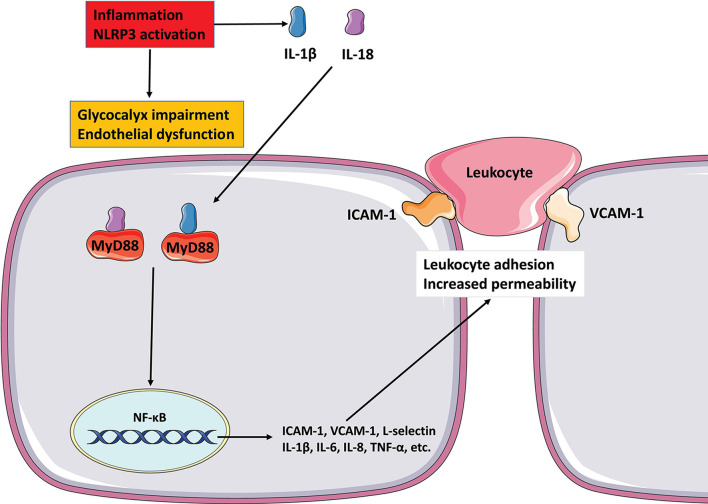
The role of glycocalyx in endothelial dysfunction and inflammation NLRP3 inflammasome activates to release IL-1 1L-18, and destroys endothelial glycocalyx. These mediators possess properties of pro-inflammatory activation. IL-1 and IL-18 binding to its cell surface receptor to activate intracellular signaling molecules MyD88, which then causes NF-κB activation. The activation of NF-κB signaling pathway increases the secretion of pro-inflammatory mediators such as cytokines and chemokines to mediate the adhesion of leukocyte and promote leukocyte extravasation. All of these reactions increase endothelial dysfunction by altering cell contractility and disrupting intercellular connections.

**FIGURE 4:**
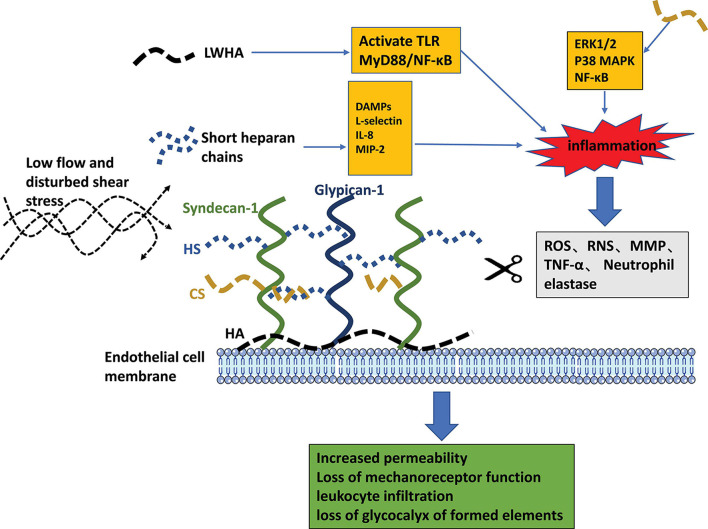
The pathogenic role of glycocalyx liberated fragments and the pathophysiologic consequences of endothelial glycocalyx loss.

In the cycle of inflammation and endothelial dysfunction, although the vascular endothelial glycocalyx layer is damaged and shed, it continues to play a critical role in regulating the development and progression of inflammation (Figures 3, 4). HS is the main component of vascular endothelial glycocalyx GAGs, which are disseminated widely on the surface and matrix of vascular cells. Numerous studies have confirmed that through its protein binding properties, HS participates in various steps of inflammation. L-selectin is constitutively expressed by leukocytes and participates in the regulation of leukocyte rolling. Thus far, no natural ligand for L-selectin has been found. In the glycocalyx, HS is known to interact with L-selectin and to act as an L-selectin ligand, regulating the rolling of leukocytes in the vascular cavity in the initial stage of inflammation (Collins and Troeberg, 2019). In the inflammatory response, a variety of transmembrane glycoproteins in the immunoglobulin superfamily, including ICAM-1 and VCAM-1, and integrins are involved in inducing leukocytes to extend and adhere tightly to the side surface of the vessel lumen. Wang (2011) found that knocking out the HS gene significantly reduced the accumulation of the chemokine IL-8 on the luminal surface of endothelial cells, while inhibiting the tight adhesion of neutrophils caused by chemokines. However, the expression levels of ICAM-1 and VCAM-1 did not change. In the same study, in the absence of any difference in endothelial permeability, transcytosis of chemokines from the tissue to the vascular cavity was greatly weakened in the HS gene knockout mice (Middleton et al., 1997). Massena et al. (2010) reported that glycocalyx HS mediate the accumulation of the chemokine MIP-2 on the surface of the endothelial cell cavity and forms a concentration gradient, mediating the movement of leukocytes toward the transmembrane site. HS can combine with chemokines to form complexes (e.g., IL-8), which increases the affinity of chemokines for corresponding receptors on the cell membrane (Koenig et al., 1998). After enzyme digestion of HS, binding of chemokines to endothelial cells is reduced, and the effect of these chemokines on vascular endothelial cells is weakened. HS can regulate leukocyte chemotaxis in many ways during the inflammatory response. These include regulating neutrophil rolling, regulating the formation of inflammation-related chemokine concentration gradients, and regulating the transport of chemokines from the inflammation site to the vascular lumen (Kumar et al., 2015).

The CS is a type of sulfated GAG and the main component of the endothelial glycocalyx layer. It is a linear polysaccharide make up of repeated disaccharide units composed of glucuronic acid and *N*-acetylhexosamine. CS is known to elicit a range of beneficial anti-inflammatory effects, including increasing type II collagen and proteoglycans, reducing bone resorption, and improving the anabolic/catabolic balance in chondrocytes (Martel-Pelletier et al., 2015). Therefore, CS is widely used in the study of osteoarthritis. Melgar-Lesmes et al. (2016) found that CS interferes with the proinflammatory activation of monocytes and endothelial cells driven by TNF-α, thus reducing the progression of inflammation and preventing the formation of atherosclerotic plaques. In this way, CS treatment might provide a new strategy for the clinical treatment of atherosclerosis. Moreover, *in vitro* studies showed that CS reduced inflammation mediators and the apoptotic process, in addition to reducing protein production of inflammatory cytokines, inducible NO synthase, and MMPs (Campo et al., 2009). The activation of NF-kB signaling is pivotal to the inflammatory response in the pathogenesis of numerous diseases. Vallières and du Souich (2010) reported benefits of CS in numerous inflammatory diseases and attributed these to a reduction in NF-kB nuclear translocation in chondrocytes and the synovial membrane. Loeser et al. (2005) reported that by reducing the phosphorylation of extracellular regulated protein kinase1/2 and p38 mitogen activated protein kinase, CS diminished the nuclear translocation of NF-kB triggered by heat shock proteins, glucose regulated proteins, fibronectin, extracellular matrix fragments, proinflammatory cytokines, IL-1β and TNF-α, Pathogen-associated molecular patterns, and lipopolysaccharides. In this way, CS reduced the expression of proinflammatory cytokines, NO synthase, cyclooxygenase 2, phospholipase A2, and MMPs and diminished the inflammatory reaction (Loeser et al., 2005). This mechanism of action of CS may explain its effect on diseases with a strong inflammatory component.

The HA belongs to a large family of GAGs and has been proven to display multiple biological functions, which depend on its molecular size (Litwiniuk et al., 2016). According to recent research, HA has anti-inflammatory properties. High-molecular weight (HMW) HA tends to be anti-inflammatory, whereas low-molecular weight (LMW) HA tends to be proinflammatory (Gall, 2010). LMW-HA can induce various proinflammatory responses, such as activation of murine alveolar macrophages (Noble et al., 1996). In addition, small HA fragments increase the expression of several cytokines, including MMP-12, plasmogen activator inhibitor-1, MIPs (MIP-1α and MIP-1β), monocyte chemoattractrant-1, keratinocyte chemoattractant, and IL-8 and IL-12 (Horton et al., 1999, 2000). Bourguignon et al. (2011) showed that LMW-HA stimulates TLR2, TLR4, and MyD88 to form a signaling complex with CD44, leading to NF-κB specific transcriptional activation and the expression of the proinflammatory cytokines IL-1β and IL-8 in a human breast cell line. Taken together, these reports suggest that LMW-HA induces inflammation via activation of TLRs and initiation of MyD88/NF-κB signaling, which leads to the production of proinflammatory cytokines and chemokines. Unlike small HA fragments, HMW-HA exhibits anti-inflammatory effects as it is a natural macromolecular polymer. Wang et al. (2006) analyzed the influence of HMW-HA on the expression of various inflammatory cytokines in patients with early-stage osteoarthritis. They reported downregulation of IL-8, inducible NO synthase, aggrecanase-2, and TNF-α gene expression in IL-1-stimulated fibroblast-like synoviocytes. Blocking the CD44 receptor with anti-CD44 antibody inhibited the downregulatory effects of HMW-HA on gene expression. Campo et al. (2011) reported that HMW-HA significantly diminished TLR4, TLR2, MyD88, and NF-kB expression and protein synthesis in synoviocytes in a murine model of osteoarthritis. They also observed reduced mRNA expression, TNFα, IL-1β, IL-17, and MMP-13 production, and inducible NO synthase gene expression in arthritic mice treated with HMW-HA (Campo et al., 2011). During inflammation, the endothelial glycocalyx is shed and degraded, and HA is degraded from a polymerized state to LMW-HA, thereby changing from an anti-inflammatory state to a proinflammatory state, which further promotes the development of inflammation. The aforementioned findings confirm the importance of protecting the integrity of the glycocalyx under inflammatory conditions.

The enzymatic degradation pathways of glycocalyx components are presented in the gray box. Pathogenic features of released fragments of short HS chains and LMWHA are depicted as orange boxes. The consequences of endothelial glycocalyx degradation are summarized in the green box.

## The Role of the Endothelial Glycocalyx Layer in Regulating the NLRP3 Inflammasome

Inflammation is a protective immune response to external stimuli pathogen-associated molecular patterns and damage-associated molecular patterns released by body damage can activate various inflammasomes (Rathinam and Fitzgerald, 2016). The NLRP3 inflammasome is one of the most comprehensively studied and is known to be involved in the development and progression of various inflammation-related diseases, such as atherosclerosis and diabetes (Danielski et al., 2020). Recent studies confirmed that the glycocalyx plays an important role in regulating the activation of the NLRP3 inflammasome. Wang et al. (2018) reported that HS inhibits inflammation by downregulating the NLRP3 inflammasome and cleavage of IL-1β during wound healing in diabetic rats. In their study, rats treated with HS exhibited decreased activation of cleaved IL-1β, IL-18, and TNF-α, as well as decreased expression of NLRP3 (Wang et al., 2018). Rajan et al. (2010) found that in a cell-free system, NLRP3 directly interacts with intrinsic RNA and HA, which was followed by activation of the NLRP3 inflammasome. These studies illustrate the important role of the glycocalyx in regulating the activation of NLRP3 inflammasomes (Rajan et al., 2010). However, the specific mechanism underlying the activity of the glycocalyx remains unclear and requires further study. It is also not known whether the glycocalyx can regulate other inflammasomes (e.g., NLRP1, NLRC4, NLRP6, and AIM2). This may be a direction for further research.

## Conclusion

Recent evidence has accumulated that endothelial glycocalyx impairment promotes a cycle of endothelial dysfunction and inflammation. The findings presented herein highlight the important role of endothelial glycocalyx integrity in combating endothelial dysfunction and vascular inflammation. Stimulation by exogenous substances or endogenous mediators of endothelial cells induces an inflammatory response, leading to endothelial glycocalyx damage and impairment of its mechanical sensory function. This leads to increased vascular permeability and changes in interactions between endothelial cells and leukocytes, which results in endothelial dysfunction and further aggravates inflammation. These changes trigger signal transduction pathways and activation of the NLRP3 inflammasome, thereby exacerbating disease. We postulate that interrupting the cycle of endothelial dysfunction and inflammation may prevent endothelial glycocalyx impairment and lead the way toward new treatments for inflammatory diseases. It is worth noting that emerging studies point to a role for statins in improving vascular dysfunction by inhibiting the NLRP3 signaling pathway and combating glycocalyx impairment. Sulodexide, a common anticoagulant and antithrombotic drug used in the clinical setting, repairs vascular endothelial cell damage, including glycocalyx impairment. It also has anti-inflammatory effects. Drugs that can combat both glycocalyx impairment and exert anti-inflammatory effects may pave the way toward new treatments for cardiovascular diseases. Another potentially interesting area of research is the possible role of the endothelial glycocalyx layer as a target in COVID-19 therapy. It is well known that COVID-19 can cause a systemic inflammatory storm and endothelial cell injury (Rovas et al., 2021). Thus, characteristics of the glycocalyx seem to be a potential target for the treatment of COVID-19.

Although our understanding of the effect of the endothelial glycocalyx layer on inflammation is growing, the specific detailed mechanism of how the glycocalyx modulates inflammation, especially under disturbed oscillatory flow conditions, remains unclear. In addition, although the glycocalyx is known to regulate not only inflammation at multiple levels but also the activation of the NLRP3 inflammasome, whether the abnormal shear stress that occurs under vascular disease conditions regulates the NLRP3 inflammasome through the glycocalyx needs to be further studied. Furthermore, whether the glycocalyx can regulate NLRP1, NLRC4, NLRP6, and AIM2 inflammasomes is not yet clear. Finally, whether both syndecans and glypicans, the two main families of glycocalyx core protein skeletons, participate in regulating the inflammatory response remains to be determined.

## Author Contributions

JQ and YC draft the manuscript. WW revised and polished the manuscript. XL and LY supervised the review and established the whole frame. All authors contributed to the article and approved the submitted version.

## Conflict of Interest

The authors declare that the research was conducted in the absence of any commercial or financial relationships that could be construed as a potential conflict of interest.

## Publisher’s Note

All claims expressed in this article are solely those of the authors and do not necessarily represent those of their affiliated organizations, or those of the publisher, the editors and the reviewers. Any product that may be evaluated in this article, or claim that may be made by its manufacturer, is not guaranteed or endorsed by the publisher.
